# Paleo-Balkan and Slavic Contributions to the Genetic Pool of Moldavians: Insights from the Y Chromosome

**DOI:** 10.1371/journal.pone.0053731

**Published:** 2013-01-16

**Authors:** Alexander Varzari, Vladimir Kharkov, Alexey G. Nikitin, Florina Raicu, Kseniya Simonova, Wolfgang Stephan, Elisabeth H. Weiss, Vadim Stepanov

**Affiliations:** 1 Phthisiopneumology Institute “Chiril Draganiuc”, Kishinev, Moldova; 2 Research Institute of Medical Genetics, Russian Academy of Medical Sciences, Tomsk, Russia; 3 Department of Biology II, Ludwig Maximilians University Munich, Planegg-Martinsried, Germany; 4 Biology Department, Grand Valley State University, Allendale, Michigan, United States of America; 5 Anthropological Research Centre “Francisc Rainer”, Romanian Academy, Bucharest, Romania; 6 Genetics Chair, Faculty of Medicine, “Carol Davila” University of Medicine and Pharmacy, Bucharest, Romania; 7 Genomnaya Diagnostika Ltd., Tomsk, Russia; Erasmus University Medical Center, The Netherlands

## Abstract

Moldova has a rich historical and cultural heritage, which may be reflected in the current genetic makeup of its population. To date, no comprehensive studies exist about the population genetic structure of modern Moldavians. To bridge this gap with respect to paternal lineages, we analyzed 37 binary and 17 multiallelic (STRs) polymorphisms on the non-recombining portion of the Y chromosome in 125 Moldavian males. In addition, 53 Ukrainians from eastern Moldova and 54 Romanians from the neighboring eastern Romania were typed using the same set of markers. In Moldavians, 19 Y chromosome haplogroups were identified, the most common being I-M423 (20.8%), R-M17* (17.6%), R-M458 (12.8%), E-v13 (8.8%), R-M269* and R-M412* (both 7.2%). In Romanians, 14 haplogroups were found including I-M423 (40.7%), R-M17* (16.7%), R-M405 (7.4%), E-v13 and R-M412* (both 5.6%). In Ukrainians, 13 haplogroups were identified including R-M17 (34.0%), I-M423 (20.8%), R-M269* (9.4%), N-M178, R-M458 and R-M73 (each 5.7%). Our results show that a significant majority of the Moldavian paternal gene pool belongs to eastern/central European and Balkan/eastern Mediterranean Y lineages. Phylogenetic and AMOVA analyses based on Y-STR loci also revealed that Moldavians are close to both eastern/central European and Balkan-Carpathian populations. The data correlate well with historical accounts and geographical location of the region and thus allow to hypothesize that extant Moldavian paternal genetic lineages arose from extensive recent admixture between genetically autochthonous populations of the Balkan-Carpathian zone and neighboring Slavic groups.

## Introduction

The Republic of Moldova is located at a geographical intersection between eastern and southeastern Europe. It shares borders with Romania to the west and Ukraine to the north, east, and south. The country is home to approximatly 4 million people, 69 percent of whom are ethnic Moldavians, with sizable minorities of Ukrainians (11%) and Russians (9%), mostly living in the eastern part of the Republic (Transnistria) and in urban areas. Moldavians speak the Moldavian language, which belongs to the Eastern Romance group of languages and is very close to the Romanian.

Modern humans began to inhabit Moldova’s territory in the Upper Paleolithic. During the Neolithic, Moldova was settled by the Starčevo–Kőrös–Criş and Linear Pottery cultures, which dominated the Balkans and central Europe in the 6^th^ millennium BC [1], [2]. As a fusion of these and other Neolithic groups, a new archaeological culture, the Cucuteni-Trypillia archeological complex, emerged by the end of the 6^th^ millennium BC. The culture lasted till ca. 2750 BC and covered a vast area extending from the Carpathian Mountains in the west to the Dnieper River in the east, and south to the shores of the Black Sea [3]. The Kurgan cultures expansion, triggered by the adaptation of pastoral nomadism by peoples in the Pontic-Caspian steppes, spread into southeastern and central Europe through the Moldovan territory in several waves over the Eneolithic and Bronze Age periods (4400−1500 BC) [4], [5]. Most likely, their arrival in central and southeastern Europe brought the Indo-European language family to Europe. It has been proposed that Paleo-Balkan tribes, the Thracians and Illyrians, originated from a mixture of indigenous peoples (Danubian farmers) and Indo-European newcomers by the end of the 2^nd^ millennium BC [6], [7].

During the Iron Age (12^th^ c. BC –4^th^ c. AD) the northern Thracian tribes, the Dacians and Getae, dominated the Carpathian Basin including the Moldovan territory [8], [9], [10]. Their closest neighbors to the east and south were nomadic and semi-nomadic tribes of Cimmerians (11^th^ - 7^th^ centuries BC), succeeded by the Scythians (7^th^ –3^rd^ centuries BC) and Sarmatians (3rd c. BC –4th c. AD). Also, Bastarnae, possibly a mixed Celto-Germanic group, settled in the southern parts of the region [11]. The Romans who had conquered the Balkans in early years AD, exerted political and cultural influence over the northern Thracians and other ethnic groups, causing their partial Romanization [8], [10], [11]. After the Roman Empire a number of groups such as Goths, Huns, Avars and Bulgars passed through the territory of Moldova, and since the 6^th^ century the Slavs had settled there and came into close economic and cultural contact with the Romanized population [10], [12]. Although the Slavic contribution to the cultural and linguistic development of Daco-Roman ethnic groups is not in doubt, the extent of their demographic contribution to indigenous populations of Moldova and Romania is not known.

From the 9^th^ until the 14^th^ century, the territory of Moldova was repeatedly invaded by different peoples from central Asia, including Magyars, Pechenegs, Cumans, Mongols and Tatars [12], [13]. After the collapse of the Mongol Empire, an influx of Vlachs (a Daco-Roman ethnic community) and Rusyns (an eastern Slavic group) to the region coincided with the formation of the Principality of Moldova (Moldavia) in the second half of the 15^th^ century [10], [13], [14]. Throughout its 500-year history the principality has been subject to the political influence of such external powers as the Kingdom of Hungary, the Grand Duchy of Lithuania and the Kingdom of Poland, as well as the Ottoman, Russian and Austro-Hungarian Empires [8]. Bessarabia, the territory in the eastern portion of the Principality, was annexed by the Russian Empire in the early 19^th^ century. The majority of the Bessarabian population continued to designate themselves as Moldavians, whereas the ethnonym “Romanian” was gaining more and more popularity throughout West Moldavia. The Bessarabian population expanded under the Russian rule, due in part to the influx of Russian and Ukrainian immigrants. The Slavic influence on Bessarabia continued through its transformation into the Moldavian Soviet Socialist Republic in 1940 and lasted until the independence of the Republic of Moldova in 1991.

Until recently, few genetic studies have been performed on Moldavians. Classical genetic markers (blood groups and serum proteins) indicate close genetic affinities of Moldavians with other southeastern European groups [15]. Similarly, autosomal Alu polymorphisms support the high degree of relatedness among southeastern European populations, including Moldavians [16]. However, these marker systems possess low-resolution power for assessing population structure. Compared with autosomal loci, Y-chromosome variation tends to exhibit a higher degree of population specificity and hence may be more informative for tracing population history [17]. Only few studies dealing with Y-chromosome diversity have been carried out on Moldavians [18], [19], [20], [21]. Furthermore, none of these previous studies focused specifically on Moldavians and their origins. Rather, they targeted larger geographic scales or other ethnic groups and were limited in their sampling and genotyping.

In this paper, we evaluated the composition of Y-chromosome lineages using the combination of 37 binary and 17 STR markers in 125 Moldavian individuals coming from two spatially separated settlements of the Republic of Moldova. For comparison, 53 Ukrainians from eastern Moldova (Transnistria) and 54 Romanians from eastern Romania were typed using the same set of markers. Comparisons to other published populations from nearby regions were made to further assess the phylogenetic context of the Moldavian Y-chromosomal pool. The results show that present-day Moldavians share their paternal ancestry with both Balkan and eastern/central European populations. This supports the historical accounts that Moldavians arose from an admixture between Vlachs and eastern Slavs.

## Materials and Methods

### Samples

**Figure 1:**
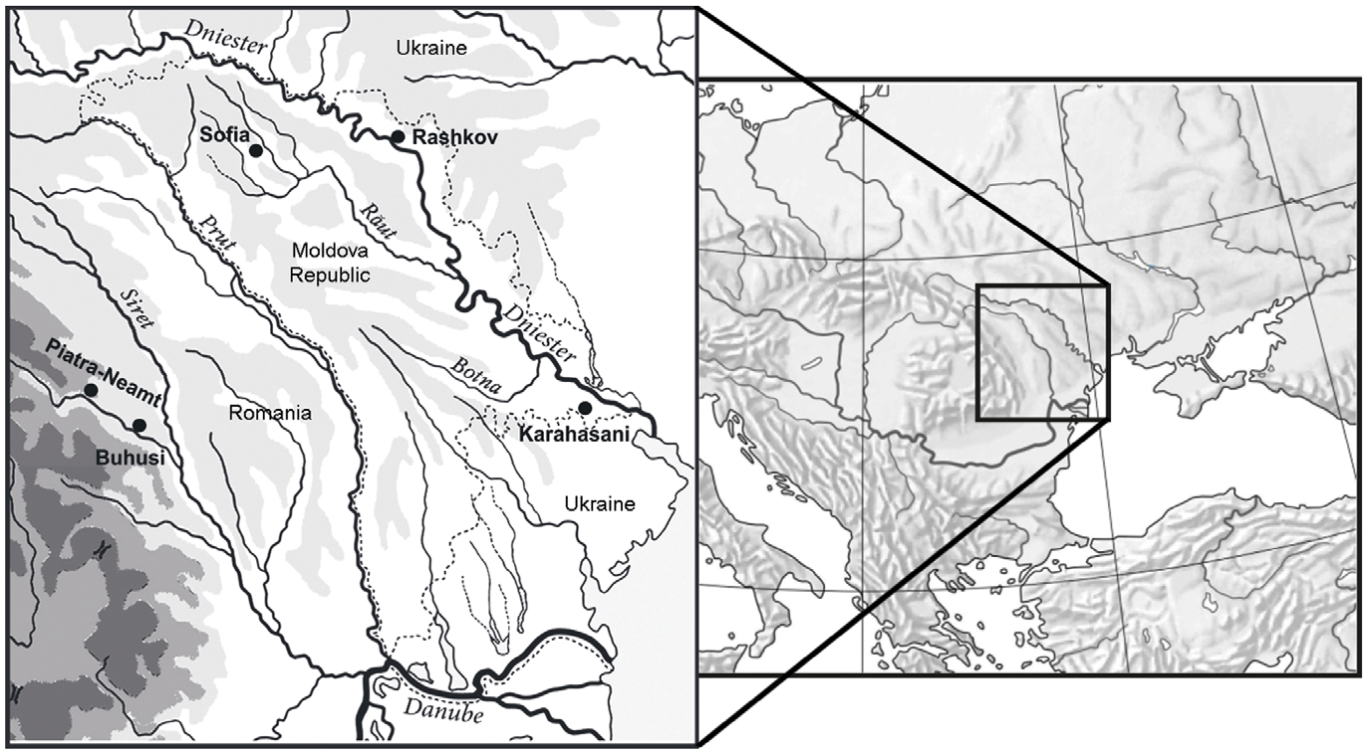
Geographical location of the samples analyzed.

A total of 232 unrelated male individuals were analyzed in the present study. The sample set comprised self-designated Moldavians from the northern settlement of Sofia and southeastern settlement of Karahasani, as well as Ukrainians from the eastern village of Rashkov in Transnistria (Republic of Moldova) and Romanians from the towns of Piatra-Neamt and Buhusi from eastern Romania (Figure 1). Taking into account the geographic profile and population size of Moldova, the two Moldavian groups sufficiently represent the whole Moldavian population. Written informed consent was obtained from all participants in the study and information about geographic and ethnic origins of their parents and grandparents was recorded. The study protocol was approved by the Ethics Committee of the Research Institute of Medical Genetics (Tomsk, Russia). Genomic DNA was extracted from peripheral blood lymphocytes using a salt-precipitation method [22].

### Genotyping

37 binary markers known to identify specific paternal lineages within Europe and West/Central Asia were analyzed in hierarchical order in agreement with the Y-chromosome phylogeny (Figure S1). 33 of these markers were typed according to previous reports, namely YAP [DYS287] [23], 12f2 [DYS11] [24], M17, M89 and DYF155S2 [25], 92R7 [26], Tat [M46] [27], M9 [28], M70, M223 and M253 [29], M52, M78 and M123 [30], P25, P37, M172, M178, M201, M207, M242 and M269 [31], SRY-2627 [32], M12, M47, M67, M92 and M267 [33], P43 [34], M73 and M458 [35], M405 [36], and M412 [37]. In addition, we genotyped four polymorphisms reported previously, namely P15 [38], v13 [39], U8 [40] and M423 [41]. Primer sequences for each of these four markers were used as previously described, or were designed by introducing a mismatched base to produce a variable restriction site in amplification products (Table 1). The deep-rooting marker M9 was typed in all samples and other markers were typed hierarchically according to their known phylogeny.

**Table 1:** PCR-RFLP protocols developed for P15, v13, U8 and M423 markers.

Marker	Primers used (5′-3′)	T^a^	Size^b^	Digestion	Fragment/s (allele)^c^
P15	F: GATTCTGGGTCTCTTCCCTTTTR: TGGGAATCACTTTTGCAACTTTCATCTGCCTTCATAC^d^	58	255	*Bst*SNI	218/37 (C) → 255 (T)
v13	F: AACAGTGGAGGACAAAGCATGTTACTGTCAGTGGC^d^R: AGTGTATCAACAATGCCCAG	58	320	*Hsp*AI	286/34 (G) → 320 (A)
U8	F: GGAGGTCTCCTGTGTCATTGR: TTATGATTTTTGGATTAAGGTTGCCATCAGGCTTTC^d^	58	311	*Bpu*14I	311 (T) → 274/37 (C)
M423	F: TTCACATTTTAACAGGGAACCAR: CAAAGGAATCACTTGGCTGTCACTTCCGATGATGC^d^	58	166	*Hsp*AI	130/36 (C) → 166 (T)

F refers to the forward primer, and R refers to the reverse primer for a particular locus.

a PCR annealing temperature in C°.

b PCR product size in base pairs.

c RFLP fragments in base pairs.

d Mismatched primer (mismatched bases are underlined).

In addition, all samples were genotyped for 17 Y-chromosome short tandem repeats (DYS19, DYS385a, DYS385b, DYS388, DYS389I, DYS389II, DYS390, DYS391, DYS392, DYS393, DYS426, DYS434, DYS435, DYS436, DYS437, DYS438, and DYS439). The primers used were described by de Knijf et al. [42] and Kayser et al. [43] to amplify DYS19, DYS389I, DYS389II, DYS390, DYS391, DYS392, DYS393, by Buttler et al. [44] to amplify DYS385a, DYS385b, DYS388, DYS426, DYS438, and by Ayub et al. [45] to amplify DYS434, DYS435, DYS436, DYS437 and DYS439. One of each primer pair was labeled with fluorescent dye TET (green), FAM (blue) or HEX (yellow). The amplified products were then pooled in two different multiplexes (DYS19, DYS389I, DYS389II, DYS390, DYS391, DYS392, and DYS393) and (DYS385a, DYS385b, DYS388, DYS426, DYS434, DYS435, DYS436, DYS437, DYS438, and DYS439) and run on ABI Prism 310 and 3130 genetic analyzers (Applied Biosystems) using GeneScan 500-TAMRA (red) as the internal standard. The alleles were named according to the number of repeat units. The number of repeat units was established through the use of sequenced reference DNA samples as suggested by de Knijf et al. [42]. Allele length for DYS389b was obtained by subtraction of the DYS389I allele length from that of DYS389II. For the duplicated microsatellite DYS385a/b, the short and long scores are reported according to allele size.

### Statistical Analysis

Statistical analyses such as haplogroup and haplotype diversity assessments, pairwise F_ST_ (for haplogroup) and R_ST_ (for haplotype) distance calculations, and AMOVA based on STR data were performed using Arlequin ver. 3.5 software [46]. The statistical significance tests for R_ST_ were performed using 1,000 permutations and for F_ST_ and AMOVA with 10,000 permutations. R_ST_ distances among compared populations were represented in two dimensions with multidimensional scaling (MDS) using the STATISTICA v.6 software package (StatSoft, Inc 1995). STATISTICA was also used to perform the principal component (PC) analysis on the covariance matrix of the Y haplogroup frequencies.

Network analysis of the STR data was carried out with the software package NETWORK version 4.6 (http://www.fluxus-technology.com). Networks were calculated by the median-joining method after having processed the data with the reduced median method [47]. To score different mutation rates upon the network construction, each STR locus was weighted proportionally to the inverse of the repeat variance.

The DYS385a and DYS385b microsatellites were not considered in genetic distance (R_ST_) and AMOVA analyses given their duplicated nature and the impossibility of assigning each allele to DYS385a or DYS385b. The DYS385a and DYS385b loci were excluded from the construction of the phylogenetic network inside haplogroup I-M423 for the same reason. However, the DYS385a and DYS385b loci were included in constructing the network of the haplogroup R-M17*, as they diverged strongly enough to always produce two distinct peaks during sequencing. Therefore both loci could be identified in all individuals carrying R-M17* chromosomes and used as two independent markers. DYS385a and DYS385b were also included in the diversity calculations, although it may have caused slight underestimates.

## Results

### Y-chromosome Haplogroup Variation

Haplogroup frequencies in the two Moldavian (separate and pooled), Romanian and Ukrainian samples are reported in Table 2. A total of 36 of the 37 genotyped binary polymorphisms were informative and defined 25 distinct haplogroups in our combined collection of Y chromosomes (Table 2, Figure S1).

**Table 2:** Sample size (N), haplogroup counts and diversity in the population groups studied.

Haplogroup name^a^	Karahasani (Moldavians)	Sofia (Moldavians)	Total Moldavians	Piatra Neamt and Buhusi (Romanians)	Rashkov (Ukrainains)
	N* = *71	N = 54	N = 125	N = 54	N = 53
E-M78*	–	2 (0.037)	2 (0.016)	1 (0.019)	–
E-v13	6 (0.085)	5 (0.093)	11 (0.088)	3 (0.056)	–
E-M123	3 (0.042)	–	3 (0.024)	–	–
G-P15*	–	1 (0.019)	1 (0.008)	2 (0.037)	–
G-U8	–	–	–	1 (0.019)	–
H-M52	–	–	–	1 (0.019)	–
I-M253	2 (0.028)	4 (0.074)	6 (0.048)	2 (0.037)	2 (0.038)
I-M423	12 (0.169)	14 (0.259)	26 (0.208)	22 (0.407)	11 (0.208)
I-M223	3 (0.042)	1 (0.019)	4 (0.032)	1 (0.019)	–
J-M267	4 (0.056)	1 (0.019)	5 (0.040)	–	1 (0.019)
J-M172*	2 (0.028)	2 (0.037)	4 (0.032)	1 (0.019)	1 (0.019)
J-M47	–	–	–	–	1 (0.019)
J-M67*	1 (0.014)	–	1 (0.008)	–	–
J-M92	–	–	–	1 (0.019)	–
J-M12	–	–	–	1 (0.019)	2 (0.038)
N-P43	1 (0.014)	–	1 (0.008)	–	–
N-M178	–	1 (0.019)	1 (0.008)	–	3 (0.057)
Q-M242	–	1 (0.019)	1 (0.008)	–	–
R-M17*	14 (0.197)	8 (0.148)	22 (0.176)	9 (0.167)	18 (0.340)
R-M458	12 (0.169)	4 (0.074)	16 (0.128)	2 (0.037)	3 (0.057)
R-M73	–	–	–	–	3 (0.057)
R-M269*	4 (0.056)	5 (0.093)	9 (0.072)	–	5 (0.094)
R-M412*	5 (0.070)	4 (0.074)	9 (0.072)	3 (0.056)	1 (0.019)
R-M405	2 (0.028)	–	2 (0.016)	4 (0.074)	2 (0.038)
T-M70	–	1 (0.019)	1 (0.008)	–	–
H± SD	0.8918±0.0167	0.8889±0.0247	0.8910±0.0128	0.8029±0.0466	0.8331±0.0363

a The phylogeny of haplogroups is shown in Figure S1.

Relative haplogroup frequencies are given within parentheses.

H, haplogroup diversity; SD, standard deviation.

In Moldavian males, 19 haplogroups were identified. The most frequent of them were I-M423 and R-M17*, comprising 20.8% and 17.6%, respectively, of all Moldavian Y-chromosomes. These were followed by haplogroups R-M458 (12.8%), E-v13 (8.8%), R-M269* and R-M412* (both 7.2%). All of the remaining lineages were present at frequencies of less than 5% in the Moldavian paternal gene pool. The haplogroup distributions were similar in the two Moldavian samples (Fisher exact test; P = 0.34011) and were in agreement with those reported previously for the Moldavian population [17], [18], [19], [20] or neighboring populations (Table S1).

In Romanians, 15 haplogroups were found. The most common Y haplogroup in this population was I-M423 (40.7%). This is the highest frequency of the I-M423 haplogroup reported so far outside of the northwest Balkans. The next most frequent among Romanian males was haplogroup R-M17* (16.7%), followed by R-M405 (7.4%), E-v13 and R-M412* (both 5.6%).

In Ukrainians, 13 haplogroups were identified. The haplogroup R-M17* was the most frequent (34.0%), followed by I-M423 (20.8%), R-M269* (9.4%), N-M178, R-M458 and R-M73 (each 5.7%). The proportions of these haplogroups were in accordance with those observed in other Slavic populations from eastern and central Europe [41], except for the R-M73 haplogroup, which is characteristic for certain central Asian populations and is almost absent in Europe [37].

No significant difference in the haplogroup frequency distribution was detected between Sofia Moldavians and other populations from the current study (P>0.05). Pairwise comparisons of Karahasani Moldavians showed significant differences between them and Romanians (P = 0.00309), as well as Ukrainians (P = 0.00432). These differences remained significant, even after applying the Bonferroni correction for multiple testing (P = 0.01854 and P = 0.02592, respectively). The Karahasani population displayed a high frequency of the R-M458 haplogroup, characteristic for eastern and central Europe. In addition, the Karahasani Moldvians showed a lower frequency of the I-M423 chromosome than the Romanians. A significant difference in Y-haplogroup composition was also detected between Romanians and Ukrainians (P = 0.00376 uncorrected for multiple comparisons and P = 0.02256 adjusted for multiple comparisons).

**Figure 2:**
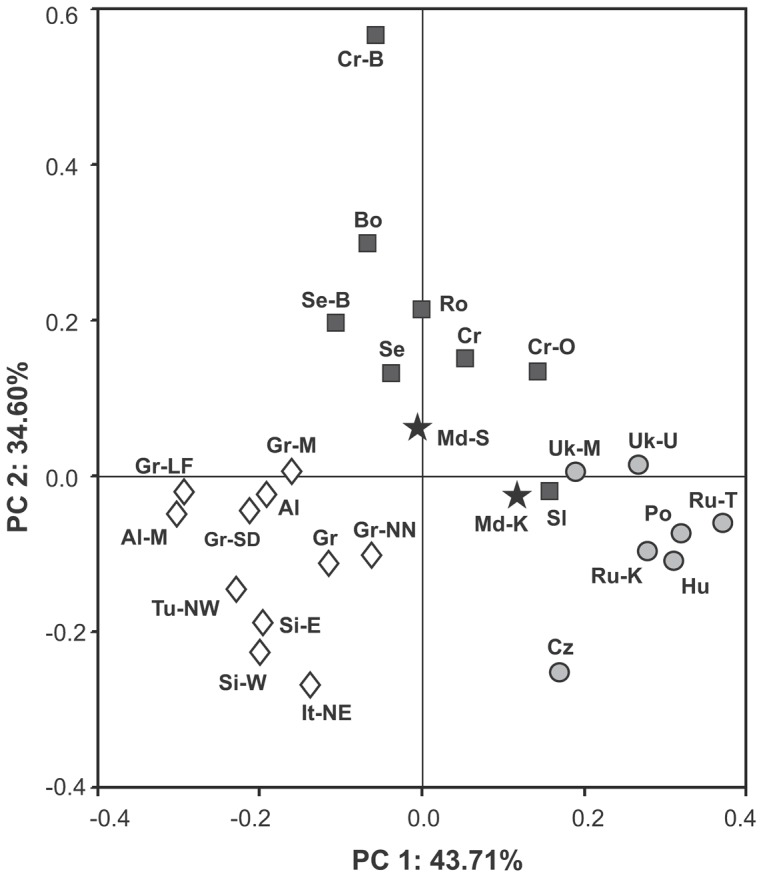
Principal Component Analysis (PCA) plot of Y chromosome haplogroup profiles showing genetic affinities among 28 populations from eastern, central and southern Europe. Original references, number of samples and name codes for all populations included in the analysis and their haplogroup frequencies are listed in Table S1. Moldavian populations are indicated by stars; north Mediterranian groups by diamonds; eastern and central European populations including Ukrainians from Moldova by circles, and north Balkan groups including Romanians by squares.

We used Principle Component (PC) analysis to compare the present data with those reported for eastern, central, and southern European populations (Table S1). These populations were included in the comparison because of their potential contribution to the genetic variability of Moldavians. To make comparisons reliable, haplogroups were rooted in the same phylogenetic level in all compared samples. Figure 2 presents the PC plot obtained. Overall, the positions of the populations in the PC plot correspond well to their assignments to specific regional groups. The first axis accounted for 43.71% of the haplogroup frequency variation and clearly separated east European populations from north Mediterranean groups. The second PC encompassed 34.60% of the observed variance and separated most of the north Balkan samples from the rest of European samples. Vector analysis (not shown) demonstrates that the north Mediterranean cluster is most associated with haplogroups J-M172, E-v13 and R-M269. The east/central European cluster was most influenced by R-M17 while the north Balkan cluster associated mostly with I-P37. The samples analyzed did not form a tight cluster on the PC plot. Indeed, Romanians clustered with samples from northwestern Balkans while the Ukrainians from Moldova fell into the east/central European cluster. Two Moldavian samples were found to occupy a more central position on the plot, with Moldavians from Sofia appearing closer to populations from southeastern Europe and Moldavians from Karahasani to populations from eastern and central Europe. These results suggest that the Moldavian Y chromosome pool is admixed, likely as a result of interactions between the Balkan and east/central European gene pools.

### Y-chromosome Haplotype Diversity

Y-STR polymorphisms were analyzed to obtain a more detailed view of Y-chromosome variation in the populations under study. Complete Y*-*chromosomal STRs haplotypes were obtained from 228 individuals, among which 181 different haplotypes were identified. In all cases but one, the chromosomes sharing a haplotype belonged to the same haplogroup. Hence, 182 compound binary-STR haplotypes were observed, among which 148 (81.3%) were individual-specific (Table S2). STR haplotype diversity for the 17-locus set ranged from 0.9895 in the Romanian population to 0.9971 in the Ukrainian population (Table S2), indicating the presence of identical haplotypes among unrelated males in all compared populations. Pairwise R_ST_ comparison between the Y-STR haplotypes (based on 15 STRs) in our samples showed that haplotype distributions were very similar in the two Moldavian samples (P = 0.28086). Yet, no significant difference was found between Moldavian and Romanian, as well as between Moldavian and Ukrainian samples. Of all pairwise comparisons, the only significant difference was observed between Romanians and Ukrainians (P = 0.01406). However, this value did not remain significant following the Bonferroni correction (P = 0.08436).

**Figure 3:**
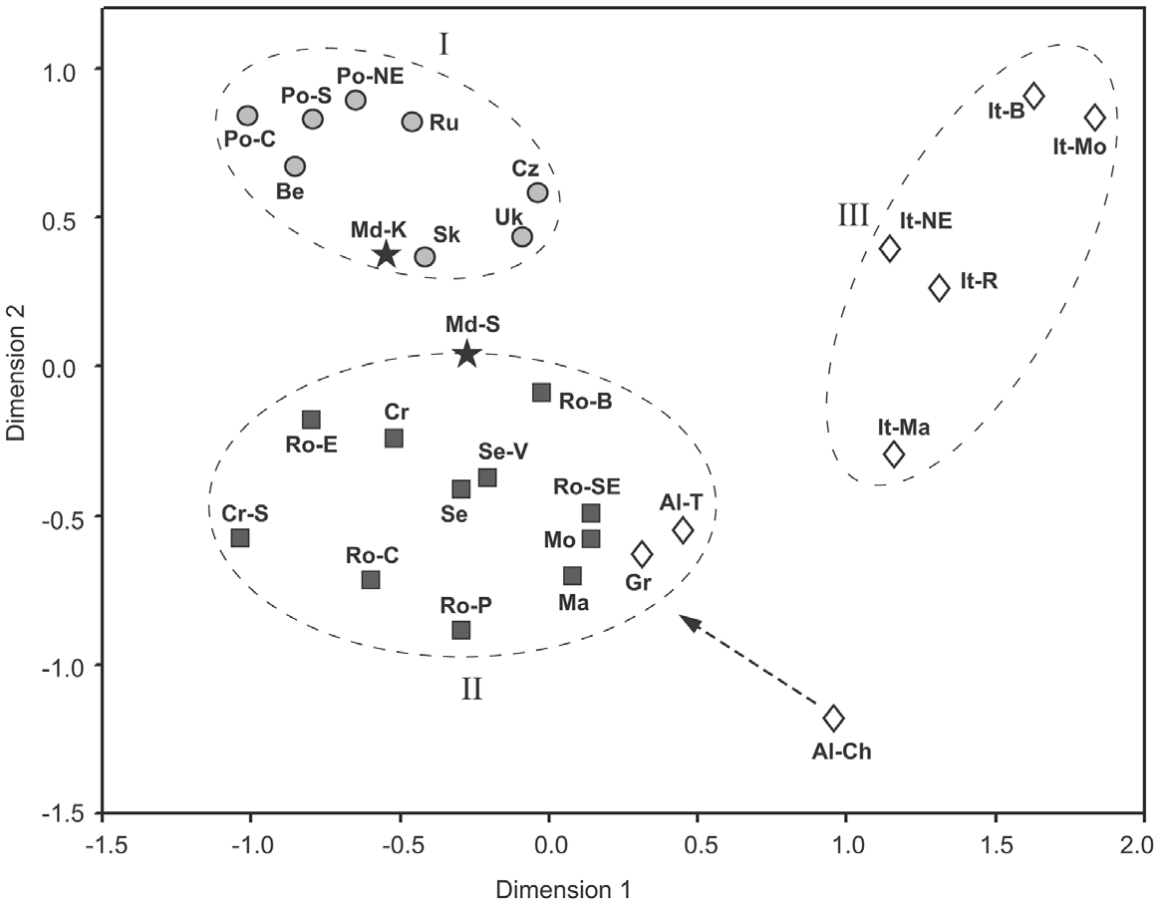
Multidimentional Scaling Analysis (MDS) plot of R_ST_ values from Y chromosome STR haplotype frequencies showing genetic affinities among 29 populations from eastern, central and southern Europe. The stress value for the MDS plot is 0.056. Original references, number of samples and haplotypes and name codes for all populations included in the analysis are listed in Table S3. Symbol designations are as in Figure 2. The ellipses are drawn around the clusters of east/central European (eastern and western Slavic) (I), southeast European (Balkan-Carpathian) (II) and Italian (III) populations. While the Cheg Albanian population is a genetic “outlier” among southeast European populations, geographically it belongs in the Balkan-Carpathian cluster (indicated by an arrow).

Once the Y-STRs were reduced to ten-locus profiles (DYS19, DYS389I, DYS389II, DYS390, DYS391, DYS392, DYS393, DYS437, DYS438, DYS439), we were able to compare our data to those from the published literature (Table S3). In the selection of populations we were guided by their relevance and importance to the population history of Moldavians. The observed haplotype diversity values in the populations studied were comparable to those found among the comparative data sets, with haplotype diversity in our Romanian sample being slightly lower. The R_ST_ genetic distances between all populations under comparison were also obtained (Table S4) and subjected to MDS analysis at two-dimensional levels. The compared populations clustered according to major geographic regions on the MDS plot (Figure 3). There is a general agreement between the MDS plot and the PC plot based on the Y-haplogroup frequencies, although the comparison was made by differing samplings of populations available for the two genetic systems. Similarly, eastern European populations formed a separate cluster, which is adjacent to the cluster encompassing the Balkan and Romanian samples. However, unlike in the PC analysis, the Italian populations were clearly separated from the south Balkan populations (Greeks and Albanians), with the latter showing greatest affinities with the north/central Balkans and Romanians. Moldavians from Karahasani and Sofia appeared to associate closely with each other along the inner margin of eastern/central European and Balkan-Carpathian clusters, respectively. Furthermore, the absence of a correlation between the ethnic and genetic diversities of the populations within the Balkan-Carpathian cluster is noteworthy. Specifically, Romanian populations appeared to be interspersed among the southern Slavic populations.

The pairwise R_ST_ comparisons showed that northern and southeastern Moldavians exhibit unequal ties to their neighbors. However, this type of phylogenetic analysis is known to be influenced by multiple-testing problems. To avoid these, AMOVA analyses were performed by assigning the populations to five groups: Moldavians, Romanians, Balkans, eastern/central Europe (eastern and western Slavs), and Italians (Table 3). Our focus was on the Moldavian and Romanian populations, due to their presumed biological connections derived from cultural similarities. The AMOVA showed no significant distinction between Moldavians and Romanians (P = 0.18851) on the one hand, as well as between the Moldavian and Romanian groups and the Balkan cluster on the other (P>0.05). Also, no significant difference was observed between the Moldavian and eastern/central Slavic groups (P = 0.62168). In fact, these differences were even less pronounced than the differences between Moldavians and Romanians (−0.11% vs. 1.66%). Noteworthy, the Romanian versus Slavic comparison revealed a significant proportion of intrapopulational differences (5.18%; P = 0.00069). The highest level of population substructure was between Italians and Moldavians (12.07%; P = 0.04525) as well as between Italians and Romanians (11.05%; P = 0.00941).

**Table 3:** AMOVA calculation results for 10 Y-STRs.

Groups^a^	Among groups	Among populations within groups	Within populations
Moldavians vs. Romanians	1.66 (P = 0.18851)	2.20 (P = 0.00020)	96.14 (P = 0.00000)
Moldavians vs. Eastern and Western Slavs	−0.11 (P = 0.62168)	1.86 (P = 0.00000)	98.24 (P = 0.00000)
Romanians vs. Eastern and Western Slavs	5.18 (P = 0.00069)	1.85 (P = 0.00000)	92.97 (P = 0.00000)
Moldavians vs. Balkans	1.57 (P = 0.16277)	3.44 (P = 0.00000)	94.99 (P = 0.00000)
Romanians vs. Balkans	−0.56 (P = 0.58693)	3.50 (P = 0.00000)	97.06 (P = 0.00000)
Moldavians vs. Italians	12.07 (P = 0.04525)	2.65 (P = 0.00000)	85.28 (P = 0.00000)
Romanians vs. Italians	11.05 (P = 0.00941)	2.73 (P = 0.00000)	86.22 (P = 0.00000)

a Twenty nine populations (the same as in MDS analysis) were pooled into five groups defined according to ethnicity (Moldavians, Romanians, Eastern and Western Slavs, Italians) or geography (Balkans) (Table S3).

**Figure 4:**
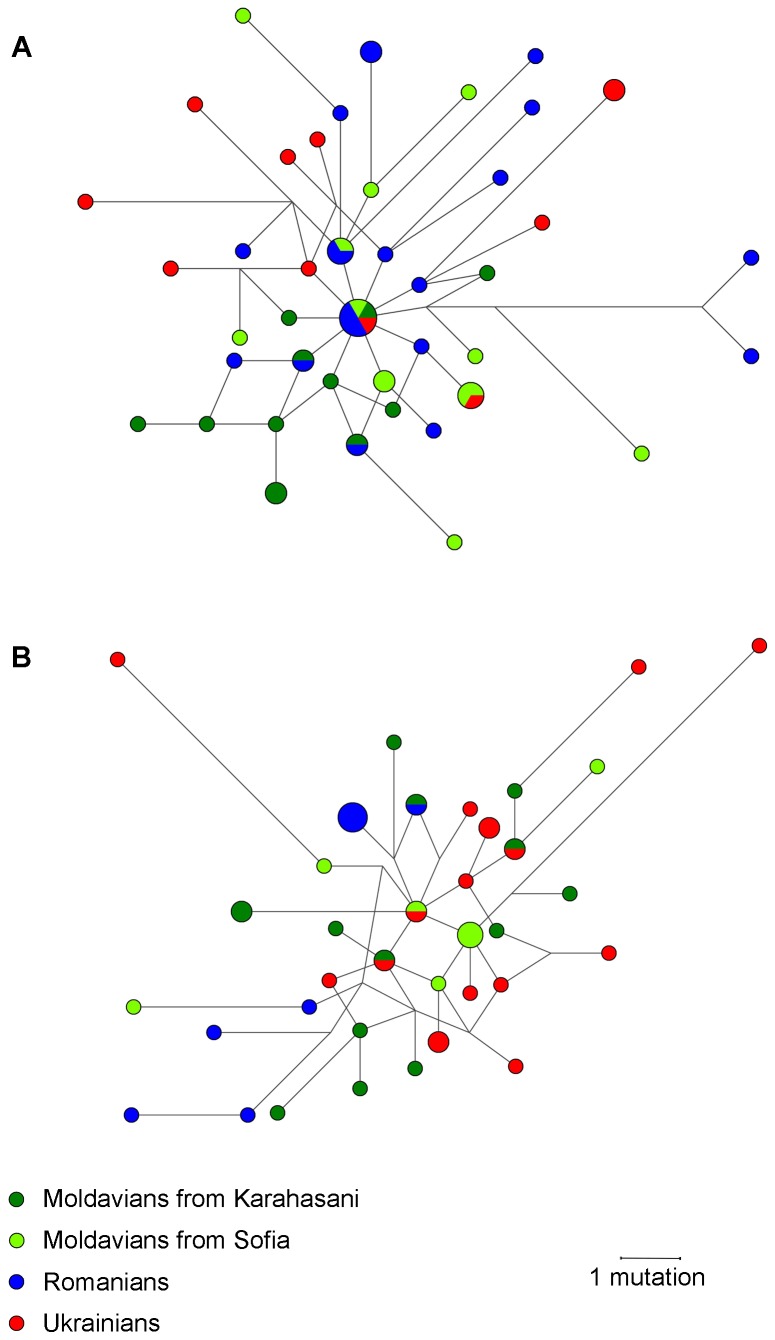
Median-joining networks showing phylogenetic relationships of the Moldavian, Romanian and Ukrainian Y-haplotypes within haplogroups I-M423 (A) and R-M17* (B). Networks were constructed from 17 STRs for haplogroup R-M17* and 15 STRs for haplogroup I-M423 as described in Materials and Methods. For each network, the smallest circles represent a count of one individual. Branch lengths are proportional to the number of mutational steps separating two haplotypes.

Haplogroups I-M423 and R-M17* were found in relatively high frequencies in Moldavians and their closest geographic neighbors, eastern Romanians and Ukrainians. In order to explore the genetic similarities of the I-M423 and R-M17* Moldavian chromosomes with those from Romanian and Ukrainian populations, median-joining networks based on 15 and 17 STRs haplotypes were generated on the background of haplogroups I-M423 and R-M17*, respectively (Figure 4). In both networks the Ukrainian and Romanian Y-STR haplotypes appeared to cluster within the respective populations. For haplogroup I-M423, Moldavian chromosomes share equal number of haplotypes with both Romanian and Ukrainian samples. In the case of R-M17*, the reduced median network of the Y-STR haplotypes indicated a closer relationship of the Moldavian Y-STR haplotypes with Ukrainian Y-STR haplotypes than with Romanian Y-STR haplotypes. Specifically, of four haplotypes shared by Moldvians with other populations three Moldavian haplotypes were found to be shared with Ukrainian haplotypes and only one haplotype was shared between Moldavians and Romanians. Pairwise R_ST_ comparisons for Y-STR haplotypes within haplogroup R-M17* further indicate that the Moldavian R-M17* chromosomes are closer related to the Ukrainian R-M17* chromosomes (R_ST_ = 0.02709; P = 0.14108) than to those of Romanians (R_ST_ = 0.20157; P = 0.0015 adjusted for multiple testing). It should be noted, however, that the total number of individuals from each population used in these analyses is small. Therefore, further study will be needed to clarify in detail the relationship of the R-M17* chromosomes in Moldavians, Romanians and Ukrainians.

## Discussion

The results of the present study show that the Moldavian paternal gene pool presents features characteristic to those found in east/central Europe and the Balkans. This is particularly supported by the following observations: (i) Moldavians display high frequencies of haplogroups I-M423 and R-M17, which are found within the populational variation characterizing paternal gene pools of southeastern and eastern Europe (Table S1); (ii) the centered position of the Moladavian samples in the space of two main components of Y-chromosomal frequencies (Figure 2); (iii) genetic distances, which place Moldavians between east/central Europe and the Balkans (Table S4; Figure 3), and (iv) the absence of significant differentiation between Moldavians and the surrounding ethno-geographical groups revealed by AMOVA analysis based on microsatellite haplotypes (Table 3).

The genetic relationship between Moldavians and Romanians deserves special attention, since these two groups speak practically the same language and share many cultural features. It is reasonable to assume that Moldavians and Romanians inherited genetic lineages, shared with other Balkan populations, from Vlachs who, in turn, received them from Paleo-Balkan tribes. However, Moldavians and Romanians do not form a cluster that would have separated them from the neighboring populations. Indeed, in the space of multi-dimensional scaling based on the R_ST_ distances between STR haplotypes, Romanian populations appeared scattered among the Balkan populations and did not cluster with the Moldavians (Figure 3). According to the AMOVA analysis, the degree of within-group differentiation among Moldavian and Romanian populations was significantly greater than genetic differences between either Romanians or Moldavians and the group comprised of the Balkan populations (Table 3). Moldavians and Romanians also appear dissimilar on the diagram of binary lineages (PC plot, Figure 2). Thus, sharing nearly the same language is not accompanied by specific genetic similarity between Moldavians and Romanians. Furthermore, Italian populations that share the Romance/Latin language with Moldavians and Romanians, show little genetic similarity with them. These results agree with previous genetic studies suggesting that the genetic landscape of southeast Europe had been formed long before the modern linguistic/ethnic landscape was shaped [16], [48].

In contrast to Romanians and most other Balkan populations, Moldavians show a clear genetic similarity to western and eastern Slavs. This is strongly implied by haplogroup R-M17, which dominates the paternal lineages of the Slavs and is broadly represented in Moldavians. Stefan et al. [18] have already noticed the increased presence of R-M17 chromosomes in Moldavians and explained it as a trait inherited from ancient (prehistoric) population of the North Pontic Steppe. However, genetic continuity in this scenario is not supported by archaeological and historical records, which suggest repeated dramatic demographic changes in Moldova’s population during the 4^th^ –14^th^ centuries AD. Recent admixture with Slavic neighbors appears to be a more parsimonious explanation for the elevated R-M17 frequency in Moldavians. The noteworthy domination of R-M17 chromosomes in Moldavians compared to Romanians is due to the R-M458 subclade. Haplogroup R-M458 likely has its roots in western/northern Poland, where it has its greatest modern concentration and microsatellite diversity [49]. Given the geographical proximity of Moldova to the Polish and other Slavic population groups and historically attested interactions between Moldavians and Slavs [10], [12], [13], [14], it is reasonable to assume that an influx of Slavs helped elevate the frequency of R-M17 chromosomes among Moldavians to underscore the Moldavian-Romanian differentiation. Furthermore, Romanians and Moldavians also display differences in the structure of R-M17* STR haplotypes. Although our network analysis (Figure 4) primarily shows homogeneity of the diversity of R-M17 haplotypes, Moldavian R-M17 chromosomes align closer with Ukrainian (Slavic) chromosomes than with Romanian ones, further supporting the contribution from Slavic neighbors to the Moldavian paternal gene pool.

Despite repeated invasions by nomads from Asian heartlands, only two (N-P43 and Q-M242) out of 125 Moldavian Y chromosomes studied here belonged to haplogroups of apparently northern/central Asian origins. These results are in good agreement with earlier studies on Y-chromosome variation in eastern and central Europe, asserting a minimal impact of gene flow from Siberia/central Asia [25], [41], [50], [51].

In conclusion, the results presented in this report allow to hypothesize that an admixture of autochthonous populations of the Balkan-Carpathian zone with neighboring Slavic populations was likely the main factor that contributed to the diversity of the Y-chromosomal genetic pool of present-day Moldavians and, in particular, to the differences in the Y chromosomal lineage composition between Moldavian and Romanian populations. Analyses of mitochondrial DNA and genome-wide assessments of haplotype sharing between Moldavians and neighboring populations would be essential to produce a comprehensive picture of phylogeographic origins of Moldavian genetic lineages.

## Supporting Information

Figure S1 **Y chromosomal haplogroups defined by the 37 binary markers used.** The solid lines represent haplogroups found in the study while the dashed lines are haplogroups not detected in the sample.
(TIF)

Table S1 **Frequencies of Y-chromosome haplogroups in the 28 populations included in the PCA.**
(XLS)

Table S2 **Y-STR haplotypes by haplogroups in the populations studied.**
(XLS)

Table S3 **Reference populations used in the MDS and AMOVA analyses.**
(XLS)

Table S4 **R_ST_ distances among 29 populations based on Y–STR haplotypes.**
(XLS)
